# Zoonotic Sporotrichosis by *Sporothrix brasiliensis* in Chile: Evidence of Emerging Transmission Under a One Health Perspective

**DOI:** 10.3390/jof12010051

**Published:** 2026-01-11

**Authors:** Patricio Godoy-Martínez, Rodrigo Muñoz, Pamela Thomson, Diego Orlandi, Flavio Queiroz-Telles, Nicomedes Valenzuela-Lopez, María Paz Villanueva, Joselin Solís, Isabel Iturrieta-González

**Affiliations:** 1 Instituto de Microbiología Clínica, Laboratorio Australomics, Universidad Austral de Chile, Las Encinas 2020, Isla Teja, Valdivia 5090000, Chile; patricio.godoy@uach.cl (P.G.-M.); maria.villanueva@uach.cl (M.P.V.); 2 Hospital Clínico de Magallanes, Av. Los Flamencos 01364, Punta Arenas 6210005, Chile; munozb30@yahoo.com (R.M.); diego.orlandi@uc.cl (D.O.); joselinsolis.lemos@gmail.com (J.S.); 3 School of Veterinary Medicine, Faculty of Life Sciences, Universidad Andrés Bello, República 440, Santiago 8370251, Chile; pamela.thomson@unab.cl; 4 One Health Institute, Faculty of Life Sciences, Universidad Andres Bello, Santiago 8370251, Chile; 5 Department of Public Health, Hospital de Clínicas, Federal University of Paraná, Curitiba 81531-990, Brazil; queiroz.telles@uol.com.br; 6 Microbiology Unit, Medical Technology Department, Faculty of Health Science, University of Antofagasta, Antofagasta 1270376, Chile; nicomedes.valenzuela@uantof.cl; 7 Department of Preclinic Sciences, Medicine Faculty, Laboratory of Infectology and Clinical Immunology, Center of Excellence in Translational Medicine-Scientific and Technological Nucleus (CEMT-BIOREN), Universidad de La Frontera, Temuco 4810296, Chile

**Keywords:** *Sporothrix brasiliensis*, zoonosis, itraconazole, lymphocutaneous infection, mycoses

## Abstract

Sporotrichosis, the most common implantation mycosis worldwide, is caused by dimorphic fungi of several species of the genus *Sporothrix*. *Sporothrix brasiliensis*, the most virulent species, has emerged in Latin America as an epi-zoonotic pathogen linked to domestic cats, dogs and humans. This report describes a confirmed human case of lymphocutaneous sporotrichosis caused by *S. brasiliensis* in Chile, associated with feline exposure in a veterinarian. Diagnosis was supported by morphological and molecular analyses of the internal transcribed spacer (ITS) and *β-tubulin* gene. The patient responded favorably to itraconazole therapy. This case highlights the growing relevance of *S. brasiliensis* in Chile and reinforces the need for integrated One Health surveillance strategies.

## 1. Introduction

Sporotrichosis is the most prevalent implantation mycosis worldwide, with a global estimate of 40,000 new cases per year [1]. This infection is caused by thermally dimorphic fungi of the genus *Sporothrix* spp., currently considered a complex composed of cryptic species [2]. Although classical transmission typically occurs via the sapronotic route, zoonotic transmission has gained increasing relevance in recent decades. *Sporothrix brasiliensis*, belonging to the *Sporothrix schenckii* species complex, is present in Brazil and in several other Latin American countries and is recognized as the most virulent species associated with domestic and feral cats [3], which act as reservoirs and sources of infection, facilitating transmission to other cats, dogs, and humans through scratches, bites, exudates, respiratory droplets, and fomites [4,5,6]. The fact that transmission can occur through contact with secretions has challenged the traditional paradigm that occurs mainly through traumatic inoculation, turning this mycosis into an emerging public health problem [7].

In Chile, the detection of *S. brasiliensis* represents a significant change in the clinical and epidemiological landscape. Although autochthonous cases of sporotrichosis caused by other species of the genus had previously been described [8,9,10], the first confirmed human isolation of *S. brasiliensis* was reported in 2023 in the Valparaíso region and was associated with feline transmission in a 59-year-old woman [11]. At the same time, an outbreak in domestic and feral felines was documented in the Magallanes Region [12], followed by other cases in the Metropolitan and Valparaíso regions, highlighting the need to implement a “One Health” approach that integrates surveillance in animals, humans, and the environment [13,14,15].

In this context, we present the clinical case of an adult patient with a confirmed diagnosis of lymphocutaneous sporotrichosis caused by *S. brasiliensis*. This case is notable both for its relevance in the context of the rapid expansion of this species and for its implications for the surveillance, treatment, and prevention of zoonotic transmission in Chile.

## 2. Case Description

On 2 October 2021 (day 0), a 28-year-old female patient from Punta Arenas, Chile, with no history of comorbidities, presented for a dermatology consultation at the Hospital Clínico de Magallanes. She was a veterinarian specializing in small animals. Her medical history reports that approximately 15 days earlier, she treated a cat with ulcerated skin lesions located on the face, neck, and extremities; the animal was a short-haired domestic cat of unknown breed that had arrived on its own at the adopters’ home; therefore, its origin was unknown. She examined the cat twice while wearing gloves and did not recall having been scratched or bitten by the cat.

**Figure 1:**
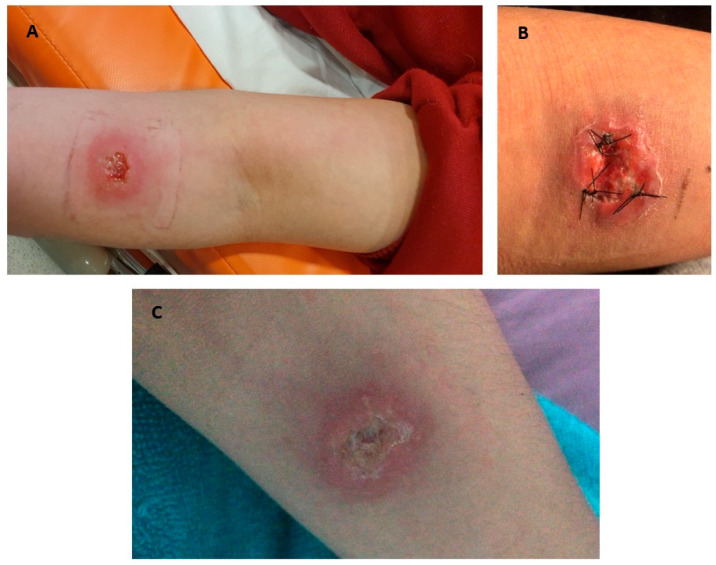
Cutaneous lesion observed in the patient before and after antifungal treatment. (**A**) Ulcerated lesion approximately 3 cm in diameter on the left forearm; (**B**) Lesion after the incisional biopsy procedure; (**C**) Clinical improvement after 30 days of itraconazole treatment.

The patient’s clinical examination revealed an ulcerated lesion on her left forearm (Figure 1A), accompanied by left axillary adenopathy and lymphangitis, associated with pain, edema, and weakness in the extremity, without paresthesia. On 7 October (day +5), the patient underwent a biopsy procedure (Figure 1B). The tissue sample was sent for mycological analysis to the Mycology Laboratory of the Institute of Clinical Microbiology and the Australomics Center, both at the Universidad Austral de Chile. While awaiting laboratory results, itraconazole 200 mg once daily (Itodal, Chile Laboratory, Macul, Chile) was prescribed for three months [16].

One month after starting treatment with itraconazole 200 mg once daily (day +35), the patient’s clinical signs had decreased, the lesion borders were smaller (Figure 1C), and the lymphadenopathy had disappeared. Upon completion of treatment, clinical recovery of the lesion was observed. No recurrence has been observed to date.

The cat was treated with itraconazole 100 mg/kg/day for four months. The animal’s condition remains unknown, as the owners did not take it for a subsequent veterinary check-up.

**Figure 2:**
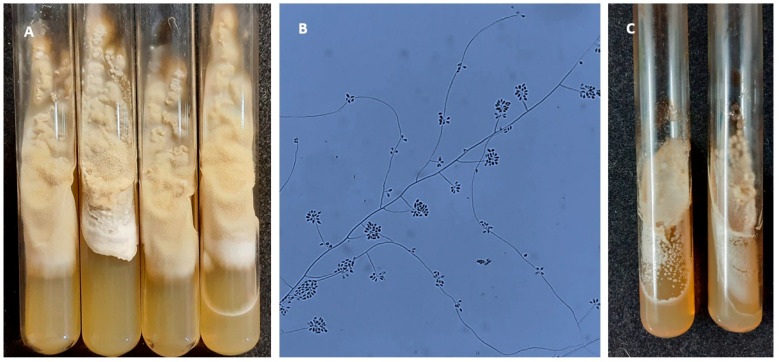
Macromorphological and micromorphological characteristics of the *S. brasiliensis* isolate obtained from the patient in the described case. (**A**) Macroscopic appearance of colonies grown on SGA after 10 days of incubation at 25 °C, whitish color, flat surface, slightly folded towards the periphery; (**B**) Thin, septate hyaline hyphae with an undifferentiated apical end from which clusters of denticulate conidia emerge, observed with lactophenol cotton blue at 1000× magnification; (**C**) Yeast-like appearance of colonies grown on SGA after incubation at 35 °C.

The biological material was inoculated onto tubes containing Sabouraud glucose agar (SGA) supplemented with chloramphenicol (0.05 g/L) and cycloheximide (0.4 g/L) (Merck, Rahway, NJ, USA) and incubated at 25 °C for 10 days. Colonies exhibiting macroscopic characteristics, such as whitish coloration, a flat, moist, glabrous surface, and slight peripheral folding (Figure 2A), together with microscopic features, including thin, septate hyaline hyphae with an undifferentiated apical end from which clusters of denticulate conidia emerge (Figure 2B), were considered suggestive of *Sporothrix* spp. [17]. These were subcultured on the same medium at 35 °C to induce conversion to yeast (Figure 2C). Subsequent DNA extraction was carried out using the ZR Fungal/Bacterial DNA MiniPrep kit (ZYMO Research, Irvine, CA, USA).

The identification of *S. brasiliensis* was supported by comparisons of two nuclear loci, i.e., the ITS region and the *β-tubulin* gene, which were amplified and sequenced using the primer pairs ITS5 (GGAAGTAAAAGTCGTAACAAGG) and ITS4 (TCCTCCGCTTATTGATATGC) [18] and Bt2a (GGTAACCAAATCGGTGCTGCTTTC) and Bt2b (ACCCTCAGTGTAGTGACCCTTGGC) [19], respectively. Sequencing was performed at Austral-omics of the Universidad Austral de Chile (Valdivia, Chile), using the ABI Prism 310 (Applied Biosystems, Foster City, CA, USA) automated sequencer. The sequences obtained were edited using SeqMan software v. 7.0.0 (DNAStar Lasergene, Madison, WI, USA) and the consensus sequences were compared in the database of the National Center for Biotechnology Information (NCBI). A sequence identity of >99% was used as the criterion to confirm correct fungal species identification.

The ITS sequence showed 100% coverage and identity with the CBS 120339 strain of *S. brasiliensis*, whereas the *β-tubulin* gene showed 99% coverage and 99.4% similarity with the IPEC43174 strain of *S. brasiliensis*. The DNA sequences of the ITS region and *β-tubulin* gene were deposited in GenBank (Table 1).

**Figure 3:**
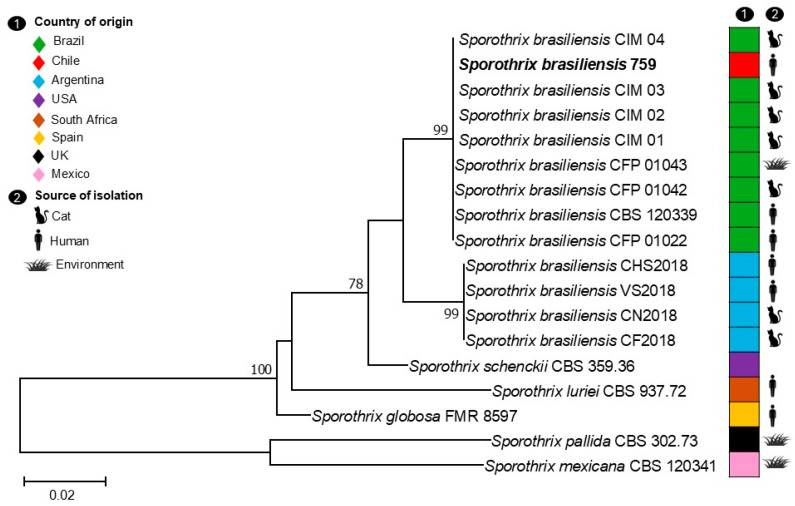
Maximum-likelihood (ML) tree constructed with ITS and *β-tubulin* sequences from 18 strains representatives of the *S. schenckii* complex clade. Bootstrap support values greater than 70% are indicated at the nodes. Country of origin and source of isolation are presented. The isolate from this study is in bold.

Phylogenetic analysis was conducted using the maximum-likelihood (ML) method with Molecular Evolutionary Genetics Analysis (MEGA) software, version 7.0. For this purpose, the *S. brasiliensis* sequences obtained in this study and other open-access sequences from Brazil and Argentina were included. In addition, sequences from other species within the *S. schenckii* complex were included. The best nucleotide substitution model determined in the same software for the combined analysis of the two phylogenetic markers was Kimura 2-parameter (K2+G). The concatenated sequence alignment, with 18 strains, comprised 805 bp (ITS 411 bp and *β-tubulin* 394 bp) with 142 variable sites (ITS 38 bp and *β-tubulin* 104 bp), of which 81 were phylogenetically informative (ITS 27 bp and *β-tubulin* 54 bp) (Figure 3).

Based on the phylogenetic analysis, two well-supported clades within the species *S. brasiliensis* were observed: one clade with 99% bootstrap support grouping Brazilian isolates, and a second subclade with 99% bootstrap support grouping Argentinian isolates. The isolate from this study (*S. brasiliensis* 759) was located in the former clade, together with other strains isolated from Brazil. The identity between the ITS sequence of the isolate *S. brasiliensis* 759 was 100% and 99.5% with Brazilian and Argentinian strains, respectively. Otherwise, the *β-tubulin* sequences showed a marked difference between the two clades, with our isolate showing 99.7% identity with Brazilian strains and 92.8% with Argentinian strains.

## 3. Discussion

We present a case of lymphocutaneous sporotrichosis caused by *S. brasiliensis* in an adult woman who had prior contact with a cat with lesions suspicious for sporotrichosis. In Chile, sporotrichosis has historically been uncommon, and cases described before 2023 were associated with infections by *S. schenckii* or *S. globosa* (within the *S. schenckii* species complex) [8,9,10]. The detection of *S. brasiliensis* in humans [11], along with its identification in domestic and feral cats in Magallanes, Santiago, and Valparaíso [12,13,14] represents an epidemiological turning point. Unlike the Brazilian outbreaks, where the infection has been associated with tropical climates and high urban feline density [20], the Chilean cases have occurred in temperate-cold climate zones, suggesting that this species exhibits remarkable ecological plasticity [21]. This adaptation phenomenon has been previously analyzed, highlighting that *S. brasiliensis* has a unique ability to persist in cold environments and become established in feline populations outside its traditional climatic distribution area [4]. A similar situation occurred in the United Kingdom, where the first three cases of cat-borne sporotrichosis caused by *S. brasiliensis* outside South America were reported. In that outbreak, the most likely source of the human infections was a 9-year-old neutered male long-haired domestic cat that had been rescued by the affected family in southeastern Brazil and brought with them to the UK three years earlier [22].

The current case shares the zoonotic nature and feline origin of the infection with other cases but differs in that the patient does not recall experiencing trauma and was wearing gloves during handling. This suggests that inoculation could have occurred through other routes, considering that previous studies have demonstrated that feline secretions can contain a high load of virulent and infective yeast forms, capable of surviving on inanimate surfaces. These findings reinforce the notion that transmission can occur even without aggressive contact or an obvious traumatic event [5].

Treatment with itraconazole 200 mg daily for three months was successful, resulting in complete resolution of the lesions with no relapses. This outcome is consistent with that reported by Rodríguez et al. [16], who observed a good response to itraconazole and terbinafine in human and feline cases with cutaneous forms. However, Brazilian studies [7] have warned that *S. brasiliensis* may exhibit greater virulence and occasionally antifungal resistance, particularly in long-standing circulating strains or in disseminated infections. Therefore, prolonged clinical follow-up and post-treatment mycological confirmation are recommended.

The detection of genetically identical strains in cats from different Chilean regions [6] points to possible dissemination through the movement of animals within the country, representing an increasingly relevant epidemiological pathway. In Argentina, Etchecopaz et al. [3] described a series of human and feline cases of *S. brasiliensis* in which genetic analysis demonstrated a close phylogenetic relationship with Brazilian strains, supporting the hypothesis of regional expansion [3]. Our phylogenetic reconstruction revealed that the *S. brasiliensis* isolate obtained in Punta Arenas (Chile) shows high genetic similarity to isolates of Brazilian origin, indicating that they share a common ancestor. This suggests that the isolate may have originated from Brazil.

In Brazil and Argentina, multiple infections have been described in veterinary personnel after contact with sick cats [5], which aligns with the present case. In the far south of Argentina, specifically in Calafate (Santa Cruz province), cases of sporotrichosis transmitted by cats have been detected. Despite the low temperatures typically associated with the far south of Argentina, this location demonstrated favorable environmental conditions for the growth of *S. brasiliensis* [3]. Recently, a study conducted in the Magallanes region (Chile) evaluated a total of 140 cats, of which five tested positives for *S. brasiliensis*; three of these animals presented clinical symptoms [6]. These findings suggest that a similar event to that observed in Argentina could be occurring in the far south of Chile.

The absence of specific biosafety measures in routine practice—such as masks, eye protection, and impermeable barriers—can facilitate exposure. Therefore, it is recommended to strengthen personal protection protocols and the management of animals with ulcerated lesions of unknown etiology [7].

The identification of *S. brasiliensis* in humans and animals in Chile highlights the urgency of implementing an integrated surveillance strategy under the “One Health” concept, as proposed previously [7,13]. This requires active collaboration between physicians, veterinarians, microbiologists, and health authorities to detect cases early, control feline outbreaks, and educate the community about preventive measures. It also underlines the need to include this emerging species in the differential diagnosis of skin lesions in patients with a history of animal contact or environmental exposure.

## 4. Conclusions

This case confirms the emergence of *S. brasiliensis* as a zoonotic agent in Chile, highlighting its capacity to adapt and spread across different geographical regions. The successful treatment with itraconazole supports its therapeutic efficacy, although continued surveillance is needed due to reports of increased virulence and antifungal resistance elsewhere. Strengthening biosafety practices and adopting a “One Health” surveillance approach are essential to prevent further transmission and ensure early detection of new cases.

## Figures and Tables

**Table 1:** Species and GenBank accession number of the *Sporothrix schenckii* complex sequences included in this study.

Species	Strain Number ^1^	Substrate	Country	GenBank Accession No ^2^.	
				ITS	*β-tubulin*
*Sporothrix brasiliensis*	CBS 120339	Human	Brazil	KP017087	AM116946
	CFP 01022	Human	Brazil	OM949881	ON014840
	CFP 01042	Cat	Brazil	MZ576444	MZ670755
	CFP 01043	Wood	Brazil	MZ576443	MZ670754
	CIM 01	Cat	Brazil	-	MH453944
	CIM 02	Cat	Brazil	-	MH453945
	CIM 03	Cat	Brazil	-	MH453947
	CIM 04	Cat	Brazil	-	MH453949
	CHS2018	Human	Argentina	MK850456	MK850452
	VS2018	Human	Argentina	MK850453	MK850449
	CN2018	Cat	Argentina	MK850455	MK850451
	CF2018	Cat	Argentina	MK850454	MK850450
	**759**	**Human**	**Chile**	**PX480145**	**PX475786**
*Sporothrix globosa*	FMR 8597	Human	Spain	FN549904	AM116964
*Sporothrix luriei*	CBS 937.72	Human	South Africa	AB128012	AM747289
*Sporohrix mexicana*	CBS 120341	Soil	Mexico	FN549906	AM498344
*Sporothrix pallida*	CBS 302.73	Soil	UK	KP017078	AM498343
*Sporothrix schenckii*	CBS 359.36	Unknown	USA	KP017100	AM116911

^1^ CBS: Culture collection of the Westerdijk Fungal Biodiversity Institute, Utrecht, the Netherlands; CFP: Collection of Pathogenic Fungi, Instituto Nacional de Infectologia Evandro Chagas, Fundação Oswaldo Cruz, Rio de Janeiro, Brazil; FMR: Facultat de Medicina i Ciències de la Salut, Reus, Spain. ^2^ ITS: Internal transcribed spacer regions of the rDNA and 5.8S region; Sequences newly generated in this study are indicated in bold.

## Data Availability

The original contributions presented in this study are included in the article. Further inquiries can be directed to the corresponding author.

## Abbreviations

The following abbreviations are used in this manuscript:

ITS	Internal Transcribed Spacer
SGA	Sabouraud Glucose Agar
NCBI	National Center for Biotechnology Information
MEGA	Molecular Evolutionary Genetics Analysis software
